# Demonstration of LanthaScreen™ TR-FRET-based nuclear receptor coactivator recruitment assay using PHERAstar, a multi-detection HTS microplate reader

**DOI:** 10.4103/0253-7613.41046

**Published:** 2008

**Authors:** Prashanth C.G. D'Souza, Nafisa Ally, Amit Agarwal, Padmaja Rani, Meenakshi Das, Dolly Sarma

**Affiliations:** Bioassay Research Laboratory, Natural Remedies Pvt Ltd, 5B Veerasandra Industrial area, Hosur Road, Bangalore - 560 100, India; 1 BMG LabTech Pty Ltd, P.O. Box 469, Mount Eliza, Victoria 3930, Australia

**Keywords:** HTS, LanthaScreen™ nuclear receptor coregulator assays, nuclear receptors, PHERAstar, time resolved-fluorescence resonance energy transfer

## Abstract

An attempt was made to demonstrate the possibility of performing LanthaScreen™ TR-FRET based nuclear receptor coactivator recruitment assay using PHERAstar, a multi-detection HTS microplate reader. LanthaScreen™ nuclear receptor coactivator recruitment assay (M/s Invitrogen corporation, USA) was performed using PPAR-gamma receptor preparation in the agonist mode. TR-FRET measurements were done on PHERAstar, a multimode microplate reader (BMG LABTECH, Germany). The Lanthascreen PPAR gamma coactivator recruitment assay was successfully performed in the PHERAstar, multimode microplate reader. This was evidenced by an assay robustness score (Z') of 0.71. The current work demonstrates the suitability of using PHERAstar, a multi-detection HTS microplate reader.for performing LanthaScreen™ TR-FRET based nuclear receptor coactivator recruitment assays.

Nuclear receptors (NRs) are a superfamily of ligand-activated transcription factors that mediate a wide range of cellular responses. The endogenous ligands for NRs include steroidal hormones, eicosanoids, retinoids, thyronines, bile acids, and other metabolites.[1] NRs influence important biological effects, such as growth, metabolism, and reproduction, and are recognized as important therapeutic targets.[2] High-throughput screening methods for nuclear receptor ligands include nuclear receptor binding assays, nuclear receptor coregulator assays, and nuclear receptor functional cell-based assays. Nuclear receptor coregulator interaction assays are an important class of cell-free functional assays. These assays are based on the principle that nuclear receptors recruit/displace coregulatory peptides upon ligand activation. One of the important assay technologies available for the study of NR coregulator recruitment is called time resolved-fluorescence resonance energy transfer[3] (TR-FRET).

In TR-FRET assays, the biomolecular interaction is detected by energy transfer between two fluorophores that are deliberately employed in these assays. The fluorescence energy transfer results in dual emissions, which can be expressed as a ratio and used as the assay signal. This ratiometric measurement is helpful to counteract assay interference like quenching exhibited by test samples. Besides the ratiometric nature of these assays, TR-FRET also utilizes the phenomenon of time-resolved fluorescence (TRF). TRF is a phenomenon exhibited by chelates of the lanthanide group of elements (europium, samarium, terbium, etc.) Upon excitation, the fluorescence that is emitted by the lanthanide chelate does not decay spontaneously like commonly employed fluorophores, instead there is delayed decay of fluorescence. Autofluorescence of test compounds/microwell plates/buffers (background fluorescence) is the bane of bioassays. The decay time of the background fluorescence falls within the decay time of commonly employed fluorophores in assay systems and thus there can be assay interference. By utilizing lanthanide chelates, the signal measurement window can be set at a much longer time interval which will be free from background autofluorescence. Thus, the TR-FRET method combines the benefits of ratiometric measurement and the TRF phenomenon.[4]

Invitrogen, USA, offers a TR-FRET-based nuclear receptor coactivator assay platform under the trade name LanthaScreen™. In the current study an attempt is made to demonstrate the possibility of performing the LanthaScreen™ nuclear receptor coregulator assay using the PHERAstar, a multilabel plate reader from BMG LABTECH, Germany. LanthaScreen™ nuclear receptor assays using the PHERAstar have not so far been reported in the literature. This demonstration would validate the compatibility of PHERAstar instrumentation for LanthaScreen™ nuclear receptor coregulator assays. This is required since advanced assay methodologies like TR-FRET require appropriate instrumentation for proper signal measurement. In the current study, the LanthaScreen™ peroxisome proliferator activating receptor-gamma (PPAR-gamma) coactivator recruitment assay (agonist mode) is employed. The assay principle is described in Figure 1.

**Figure 1:**
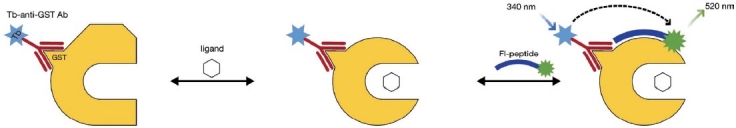
Principle of the nuclear receptor (NR) agonist-dependent coactivator peptide recruitment assay: Tb-anti-GST antibody indirectly labels the nuclear receptor by binding to the GST tag. Binding of the agonist to the NR causes a conformational change that results in an increase in the affinity of the NR for a coactivator peptide. The close proximity of the fluorescently labeled coactivator peptide to the terbium-labeled antibody causes an increase in the TR-FRET signal. (Reproduced with permission from Invitrogen Corporation, USA)

In brief, the assay procedure was as per Invitrogen's kit insert (catalog # PV 4548). All the reagents were procured from Invitrogen, except rosiglitazone, which was obtained from Cayman Chemicals, USA. The assay was performed in 384 black microwell plates (Corning # 3676). A 20 μl total assay reaction included 5 nM GST-tagged PPAR-gamma receptor (glutathione *S* transferase-tagged PPAR-gamma receptor) 125 nM of coregulator peptide, 5 nM of TB-anti-GST-tagged antibody (terbium-anti-glutathione *S* transferase tagged), 5 mM DTT (dithiothreitol), and varying concentrations of rosiglitazone (a known agonist for PPAR gamma receptor) in the assay buffer supplied by Invitrogen. The negative control was devoid of the agonist but contained everything else contained in the agonist wells. Following 1-h incubation in the dark, TR-FRET measurements were made in the PHERAstar using the following settings: optical module - LanthaScreen™, delay time - 100 μsec, and integration time - 200 μsec. The ratiometric emission (520/490) was plotted against varying ligand concentrations. The data was analyzed using GraphPad Prism, version 4.00 for Windows (GraphPad Software, San Diego, California, USA) using the sigmoidal curve equation with variable slope to obtain EC_50_ values [Figure 2]. The assay quality/robustness score - Z' was calculated as per Zhang[5] *et al*. and was found to be 0.71 (a value above 0.5 indicates a very robust assay). From the observed data, the PHERAstar, a multilabel plate reader from BMG Labtech, Germany, is suitable for LanthaScreen™ nuclear receptor coregulator assays.

**Figure 2:**
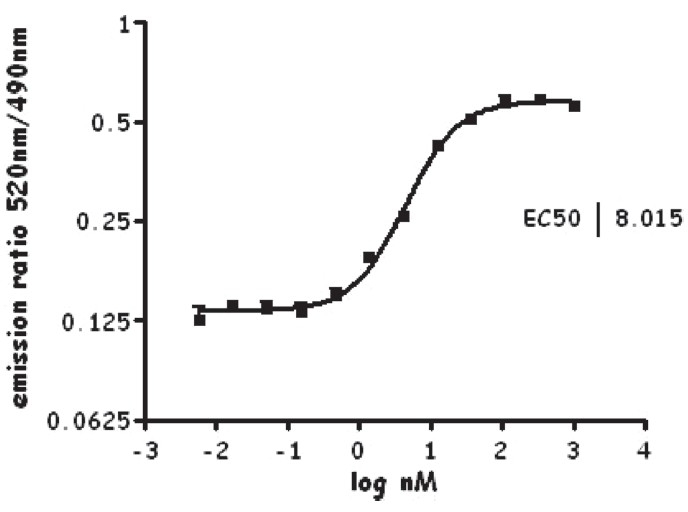
Effect of rosiglitazone in LanthaScreen™ TR-FRET PPARgamma coactivator assay in the agonist mode. Mean ± SEM (*n* = 4). The reaction mixture contained 5 nM GST-tagged PPAR gamma receptor, 125 nM of coregulator peptide, 5 nM of TB-anti-GST-tagged antibody, 5 mM DTT (dithiothreitol), and varying concentrations of rosiglitazone (1000-0.0056 nM) Following 1-h incubation in the dark, TR-FRET measurements were made in the PHERAstar multilabel plate reader
